# Antimicrobial and Anti-Inflammatory Activities of MAF-1-Derived Antimicrobial Peptide Mt6 and Its D-Enantiomer D-Mt6 against Acinetobacter baumannii by Targeting Cell Membranes and Lipopolysaccharide Interaction

**DOI:** 10.1128/spectrum.01312-22

**Published:** 2022-10-03

**Authors:** Delong Kong, Xuan Hua, Rui Zhou, Jie Cui, Tao Wang, Fanyun Kong, Hongjuan You, Xiangye Liu, Joseph Adu-Amankwaah, Guo Guo, Kuiyang Zheng, Jianwei Wu, Renxian Tang

**Affiliations:** a Jiangsu Key Laboratory of Immunity and Metabolism, Department of Pathogenic Biology and Immunology, Xuzhou Medical University, Xuzhou, China; b Department of Physiology, Xuzhou Medical University, Xuzhou, China; c National Demonstration Center for Experimental Basic Medical Sciences Education, Xuzhou Medical University, Xuzhou, China; d School of Basic Medical Sciences, Guizhou Medical University, Guiyang, China; e Key Laboratory of Medical Microbiology and Parasitology of Education Department of Guizhou, Guizhou Medical University, Guiyang, Guizhou, China; Ohio State University

**Keywords:** antimicrobial peptides, anti-inflammation, *Acinetobacter baumannii*, LPS, SEM

## Abstract

Antibiotic resistance in Acinetobacter baumannii is on the rise around the world, highlighting the urgent need for novel antimicrobial drugs. Antimicrobial peptides (AMPs) contribute to effective protection against infections by pathogens, making them the most promising options for next-generation antibiotics. Here, we report two designed, cationic, antimicrobial-derived peptides: Mt6, and its dextroisomer D-Mt6, belonging to the analogs of MAF-1, which is isolated from the instar larvae of houseflies. Both Mt6 and D-Mt6 have a broad-spectrum antimicrobial activity that is accompanied by strong antibacterial activities, especially against *A. baumannii* planktonic bacteria and biofilms. Additionally, the effect of D-Mt6 against *A. baumannii* is stable in a variety of physiological settings, including enzyme, salt ion, and hydrogen ion environments. Importantly, D-Mt6 cleans the bacteria on Caenorhabditis elegans without causing apparent toxicity and exhibits good activity *in vivo*. Both Mt6 and D-Mt6 demonstrated synergistic or additive capabilities with traditional antibiotics against *A. baumannii*, demonstrating their characteristics as potential complements to combination therapy. Scanning electron microscopy (SEM) and laser scanning confocal microscope (LSCM) experiments revealed that two analogs displayed rapid bactericidal activity by destroying cell membrane integrity. Furthermore, in lipopolysaccharide (LPS)-stimulated macrophage cells, these AMPs drastically decreased IL-1β and TNF-a gene expression and protein secretion, implying anti-inflammatory characteristics. This trait is likely due to its dual function of directly binding LPS and inhibiting the LPS-activated mitogen-activated protein kinase (MAPK) signaling pathways in macrophages. Our findings suggested that D-Mt6 could be further developed as a novel antimicrobial/anti-inflammatory agent and used in the treatment of A. baumannii infections.

**IMPORTANCE** Around 700,000 people worldwide die each year from antibiotic-resistant pathogens. Acinetobacter baumannii in clinical specimens increases year by year, and it is developing a strong resistance to clinical drugs, which is resulting in *A. baumannii* becoming the main opportunistic pathogen. Antimicrobial peptides show great potential as new antibacterial drugs that can replace traditional antibiotics. In our study, Mt6 and D-Mt6, two new antimicrobial peptides, were designed based on a natural peptide that we first discovered in the hemlymphocytes of housefly larvae. Both Mt6 and D-Mt6 showed broad-spectrum antimicrobial activity, especially against *A. baumannii*, by damaging membrane integrity. Moreover, D-Mt6 showed better immunoregulatory activity against LPS induced inflammation through its LPS-neutralizing and suppression on MAPK signaling. This study suggested that D-Mt6 is a promising candidate drug as a derived peptide against A. baumannii.

The resistance of microorganisms to currently available antimicrobials has proven to be a serious public health issue (1). Antibiotic-resistant infections kill approximately 5.3 million people worldwide each year, and among them, hospital-acquired infections induced by drug-resistant pathogens are of great concern (2). It has been estimated that if effective strategies do not counteract these hospital-acquired infections, around 10 million deaths annually will be caused by the infections by 2050 (3). Hence, there is a pressing need to find and develop novel medications with better pharmacological profiles and fewer side effects (4–6). Acinetobacter baumannii is one of the multidrug-resistant pathogens that causes hospital-acquired infections. Numerous clinical studies are slowly revealing that it is difficult to cure A. baumannii infections due to its serious drug resistance and the side effects of anti-A. baumannii drugs (7–11). As a result, the World Health Organization has designated it as a top priority for novel antibiotic development (12).

Most antibacterial targets are present in the cytoplasm of a bacterium. However, A. baumannii could reduce the cytoplasmic drug concentration due to its efflux pumps, porins, and antibiotic-impermeable membranes as a Gram-negative bacterium (13). Moreover, a large amount of lipopolysaccharide (LPS) released by the bacteria could stimulate the body's immune system and trigger an inflammatory response. The excessive inflammatory response will further aggravate the development of the disease, induce a pathogen infection, and even cause endotoxin shock, sepsis, nosocomial pneumonia, etc. (14). Therefore, a new anti-*A. baumannii* drug that possesses antibacterial activity and can inhibit the inflammatory response caused by LPS would be more conducive to increasing its clinical practicability.

Due to their strong antibacterial action, antimicrobial peptides (AMPs) have emerged as new, promising drugs for the topical and systemic therapy of infectious diseases (15, 16). In addition, Several AMPs have been proven to protect against biofilm development during bacterial colonization (17–19). Moreover, some cationic peptides exert immunomodulatory effects by neutralizing the ability of LPS to produce inflammatory cytokines (20–23).

Musca domestica antifungal peptide-1 (MAF-1, GENBANK: HM178948) is a new cationic AMP that was isolated from housefly instar larvae and displays outstanding antibacterial activity (24). Our earlier studies demonstrated that MAF-1A derived from MAF-1 displays strong and rapid antimicrobial activities (25, 26). However, the stability of MAF-1A is poor, which greatly limits the clinical application of MAF-1A as a drug screening molecule. Studies have found that AMPs could improve their stability and cell selectivity while maintaining high antibacterial activity by substituting different amino acids or dextral (D) modification (27). Furthermore, some natural AMPs under proper modification have been developed into commercial drugs or as candidates for new antibacterial drugs by which to treat disease. For instance, the well-studied human cathelicidin LL-37 and its analog attenuate the migration of neutrophils and improve the survival of mice with sepsis-induced acute lung injuries (28). Marine peptide-N6NH2 and its derivative possess strong activity against intracellular bacterial infections, while NP213 is a novel onychomycosis therapy candidate for fungal infections (29, 30). Therefore, the antibacterial peptides based on MAF-1A need to be further optimized and improved.

To develop new antibiotic molecules against A. baumannii with high antibacterial activity and excellent stability, in the current study, we designed and synthesized two derived peptides, Mt6 and D-Mt6, as MAF-1A analogs by employing amino acid substitutions and d-amino acid modifications. Furthermore, we studied the effects of the analogs on biofilm formation and the mechanisms of the analogs against A. baumannii. Meanwhile, the antimicrobial activity of Mt6 and D-Mt6 was evaluated *in vivo* with a Caenorhabditis elegans model that was infected with A. baumannii. Finally, the immunoregulatory activity of the two analogs in targeting LPS was assessed. We concluded that a d-amino acid modification-based design could be applied to AMPs to improve their molecular stability while retaining their antibacterial activity, compared to their progenitor. In addition, the successful modifications based on MAF-1A generate a broad-spectrum antibiotic activity with a particular efficacy against A. baumannii infections and LPS-induced inflammation.

## RESULTS

### Design of the MAF-1A analogues.

**FIG 1:**
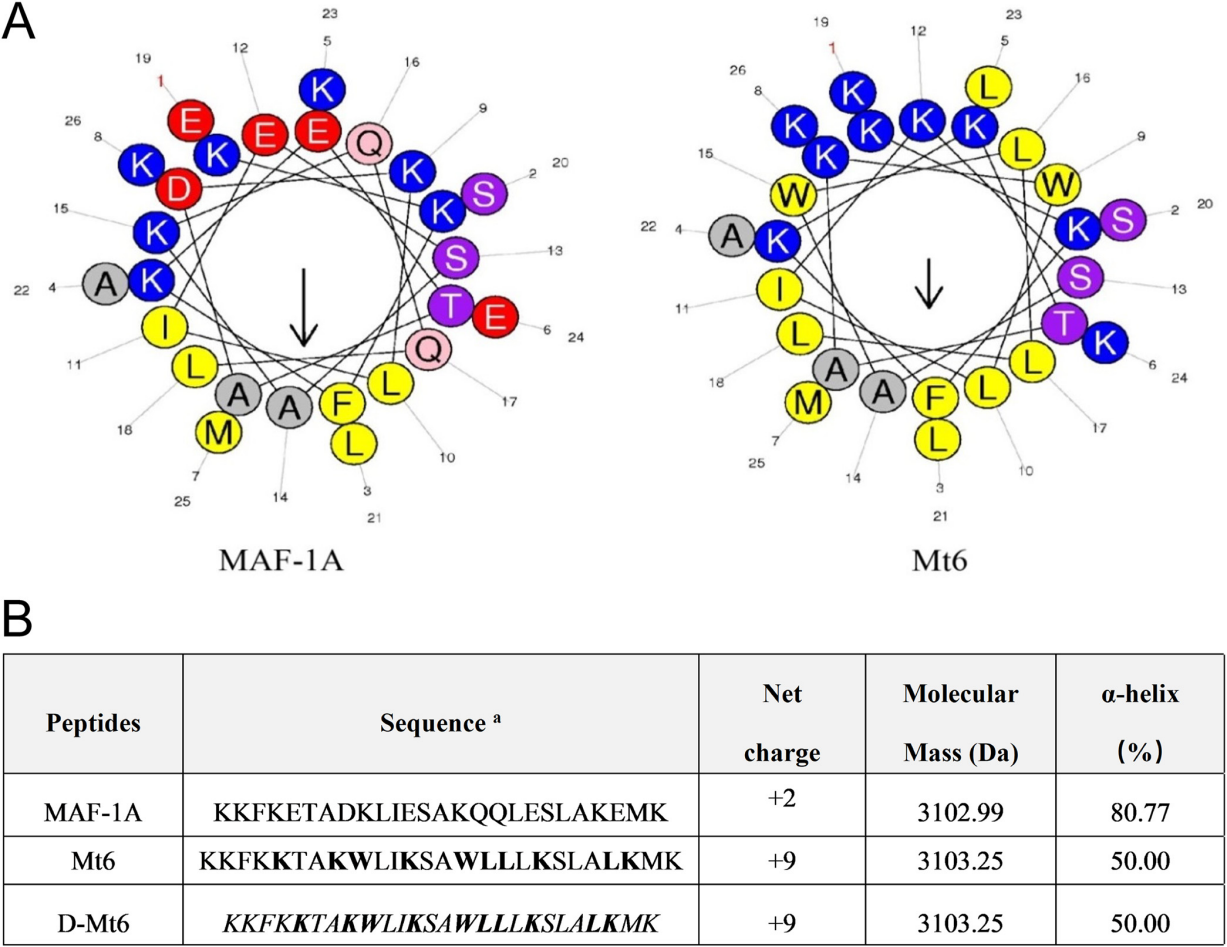
Characteristics of MAF-1A derived peptides. (A) Helical diagrams for MAF-1A and Mt6. Arrows indicate the direction of the hydrophobic moment. The helical wheel was generated using Heliquest: http://heliquest.ipmc.cnrs.fr/cgi-bin/ComputParams.py. Note: blue is a polar positively charged amino acid; red is the polarity of negatively charged amino acids; pink and purple are polar uncharged amino acids; yellow and gray are nonpolar amino acids. (B) Amino acid sequences and physical characteristics for MAF-1A and its analogs. Bold letters are substituted amino acids; italics represent d-substitution.

To enhance the cationicity of MAF-1A, the analog Mt6 was designed and synthesized with the positively charged alkaline amino acid *Lys* substituted for the acidic amino acids *Glu* (5, 12, 19, 24) and *Asp* (8). These substitutions increased the net charge of Mt6 from +2 (MAF-1A) to +9. *Gln* (16, 17) amino acids were replaced by *Leu* to increase the hydrophobicity. *Lys* (9, 15) amino acids were replaced by *Trp* with large indole side chains to enhance the ability of the derived peptide to penetrate the cell membrane. Consistently, a helix-wheel analysis revealed the distribution of hydrophobic and polar or hydrophilic amino acids in the sequences of those peptides (Fig. 1A). The analog D-Mt6 is obtained via the d-amino acid substitution of all of the amino acids of Mt6. Fig. 1B shows the sequences, the calculated molecular masses, and the net charges of the peptides. Many AMPs that target the cell membrane employ this conformation, which is recognized as closely related to their antibacterial function. Hence, the two analogues could exert antimicrobial activity as strong candidates for new membrane-disruptor peptides.

### The analogs of MAF-1A show a wide antibacterial spectrum and strong anti-Acinetobacter baumannii activity.

To assess the activities against pathogens, a standard minimum growth inhibitory concentrations (MIC) assay was carried out. Table 1 summarizes the peptide doses required to suppress planktonic bacteria and fungus. Mt6 and D-Mt6 showed different degrees of inhibitory activity against Gram-positive bacteria (S. aureus and B. subtilis) and Gram-negative bacteria (E. coli, K. pneumoniae, P. aeruginosa, A. baumannii). This indicates that the enhanced anti-Candida albicans activity and the wider antibacterial spectrum were possessed by the two analogues, compared with MAF-1A. Significantly, the two derived peptides both showed strong antibacterial activity against A. baumannii.

**TABLE 1:** *In vitro* susceptibility of G+ bacteria, G− bacteria and fungus to analog peptides^*a*^

Peptides	MIC^*b*^ (μg/mL)						
	C. albicans	S. aureus	B. subtilis	E. coli	K. pneumoniae	P. aeruginosa	A. baumannii
MAF-1A	600	>1,200	>1,200	>1,200	>1,200	>1,200	>1,200
Mt6	192	256	256	16	8	32	8
D-Mt6	128	>256	128	8	8	16	8
Polymyxin B	-^*c*^	-	-	2	2	8	2

a The values are the means of three independent experiments performed in four replicates.

b MIC was defined as the minimal peptide concentration required for the total inhibition of microbe growth in a liquid medium.

c -, Not measured.

### The derived peptides exhibit good stability against A. baumannii.

AMPs can lose their activity due to many factors in the physiological environment, which greatly limits their clinical application. Given this, we determined the MIC values of Mt6 and D-Mt6 on A. baumannii in different salt ion solutions, hydrogen ion solutions, and enzyme solutions. As shown in Table 2, the antibacterial activity of Mt6 and D-Mt6 was either a partial effect or a nonexistent effect in separate 4.5 mM KCl, 150mM NaCl, 1mM MgCl_2_, or different pH-valued environments. Furthermore, in the presence of enzymes, D-Mt6 retained a MIC of 8 μg/mL while the MIC value of Mt6 was increased in both 0.15 mg/mL and 0.6 mg/mL trypsin solutions. These results indicated that the derived peptides exhibited good stability against A. baumannii. Moreover, D-Mt6 was more stable than Mt6 in different physiological conditions resembling *in vivo* environments.

**TABLE 2:** MIC values (μg/mL) of analog peptides in various physiological environments against A. baumannii

Peptides	MIC(μg/mL）								
	Control	150 mM NaCl	4.5 mM KCl	1 mM MgCl_2_	0.15 mg/mL trypsin	0.6 mg/mL trypsin	pH 5.45	pH 7.45	pH 9.45
Mt6	8	16	8	16	>256	>256	8	8	8
D-Mt6	8	16	8	16	8	8	8	8	8

### Derived peptides kill A. baumannii by permeabilizing the bacterial membrane.

**FIG 2:**
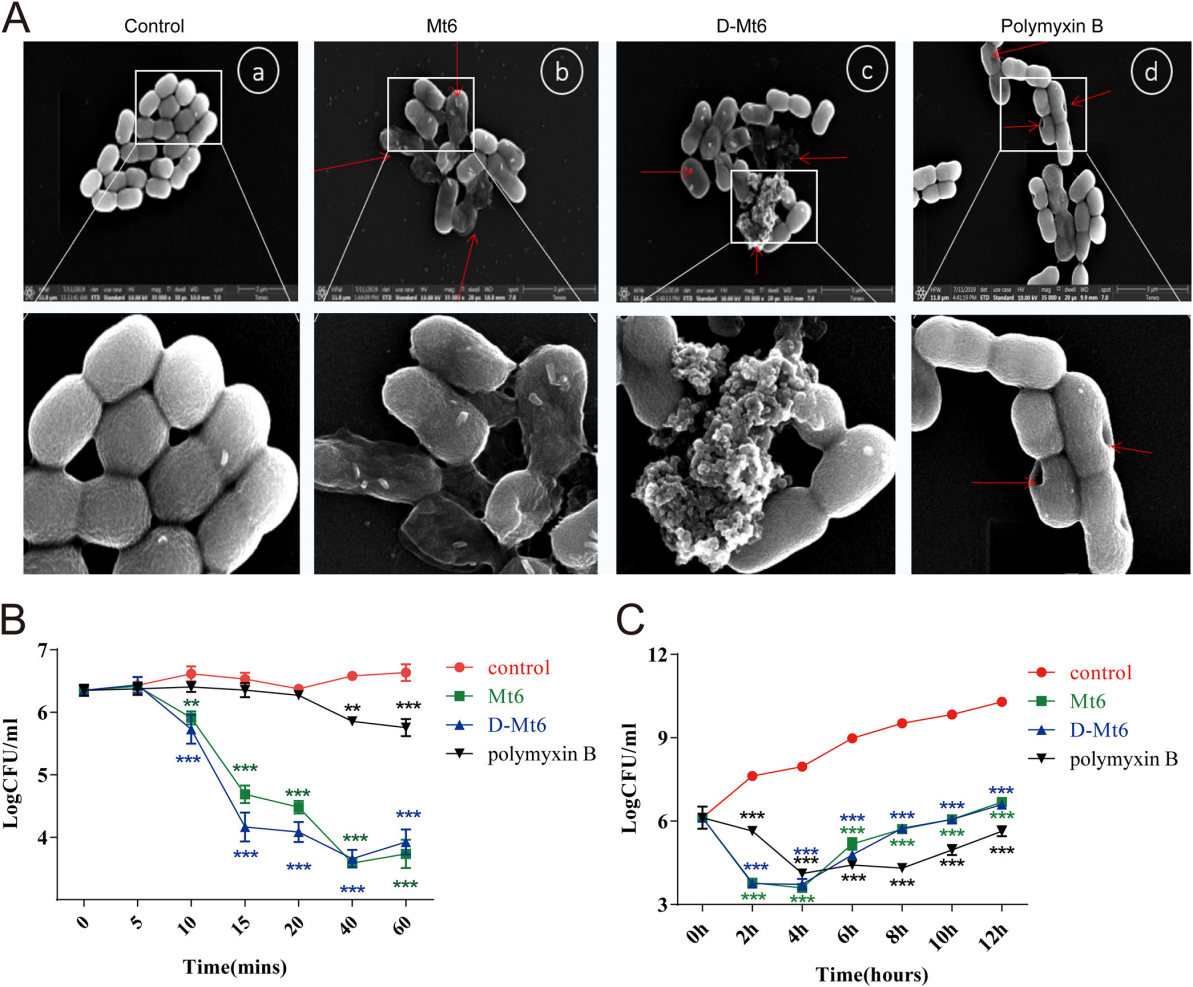
Antimicrobial activity and mechanism of derived peptides. (A) Scanning electron microscopy of A. baumannii control (A), treated with Mt6 (B), treated with D-Mt6 (C), and polymyxin B (D). The broken membranes and flowed red arrows indicate out-intracellular inclusions. (B) Time-kill curves performed on A. baumannii treated with Mt6/D-Mt6 and polymyxin B in 1 h under the MIC. (C) Time-kill curves performed on A. baumannii treated with Mt6/D-Mt6 and polymyxin B in 12 h under the MIC. All of the experiments in these panels were performed in triplicate. Data are expressed as the mean ± standard deviation of three independent experiments. **, *P* < 0.01; ***, *P* < 0.001 versus a control without peptide.

To assess the possible mechanism of the action of Mt6 and D-Mt6 on bacteria, scanning electron microscopy (SEM) was used to examine the membrane morphology. As depicted in Fig. 2A, compared with those untreated with the peptides, A. baumannii treated with the peptides revealed a significant difference in membrane morphology. D-Mt6 triggered the disruption of the A. baumannii plasma membrane and the release of plasmic contents, which led to the bacteria losing their original cellular structures. In contrast, the surface morphology of untreated A. baumannii cells was smooth, and displayed no apparent deviation in the cell surface. Meanwhile, the plasma membrane of the positive-control drug polymyxin B-treated A. baumannii cells remained relatively intact, with smooth surfaces and only partial pores formed. These results indicated that D-Mt6 treatment caused much more severe damage to A. baumannii membranes. The fluorescent dye PI is usually blocked outside an intact cell membrane, whereas it can penetrate a damaged cell membrane and insert nucleic acid (31). The permeabilization of the A. baumannii membrane induced by Mt6 and D-Mt6 was further verified by laser scanning confocal microscopy of the PI influx, as depicted in Fig. S1. Furthermore, we dynamically monitored the bactericidal effects of Mt6 and D-Mt6 on A. baumannii over a short time (1 h) and a long time (12 h) at the MIC. As is shown in Fig. 2B and C, both Mt-6 and D-Mt6 began sterilize A. baumannii within 10 min at the MIC, which was significantly faster than that observed with the positive-control, polymyxin B. Meanwhile, Mt6 and D-Mt6 could provide the same long-term antibacterial effect as polymyxin B. Collectively, these results suggest that both Mt6 and D-Mt6 display powerful and rapid antibacterial properties by permeabilizing the bacterial membrane.

### Antibacterial activity in combination with traditional antibiotics.

**FIG 3:**
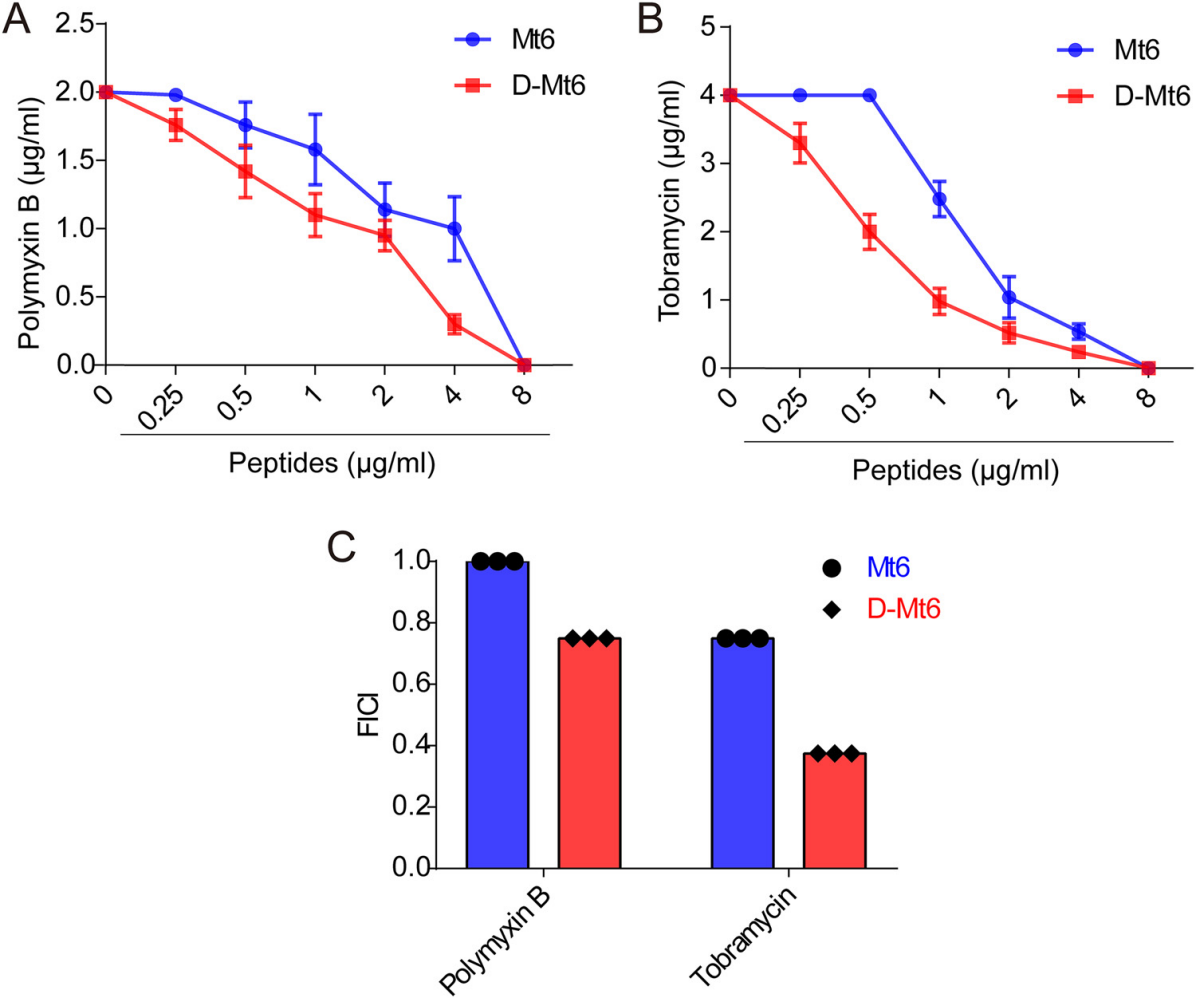
Interaction of derived peptides with polymyxin B or tobramycin tested on A. baumannii. (A) The efficacies of combinations of Mt6 or D-Mt6 with polymyxin B were observed by checkerboard titration. (B) The efficacies of combinations of Mt6 or D-Mt6 with tobramycin were observed by checkerboard titration. The data points of the isobolograms represent the concentrations of the two individual compounds in different combinations that lead to complete bacterial growth inhibition. The calculated FICIs are displayed in (C). FICI: fractional inhibitory concentration index. FICI ≤ 0.5 indicates synergy, and 0.5 < FICI ≤1 indicates an additive effect.

Checkerboard titration experiments were performed to assess the combined effect of peptides with polymyxin B or tobramycin. The results showed 4-fold lower MIC values for D-Mt6 when tested in the presence of half-MICs of polymyxin B (Fig. 3A) and 8-fold lower MIC values in the presence of half-MICs of tobramycin (Fig. 3C) compared to that of D-Mt6 alone. A harmonious interaction between D-Mt6 and tobramycin (fractional inhibitory concentration index [FICI] = 0.375) was indicated in addition to an additive effect in combination with polymyxin B (FICI = 0.75). Additionally, Mt6 also showed an additive effect when combined with tobramycin and polymyxin B, respectively (Fig. 3C).

### D-Mt6 exhibits biofilm inhibition and eradication activities.

**FIG 4:**
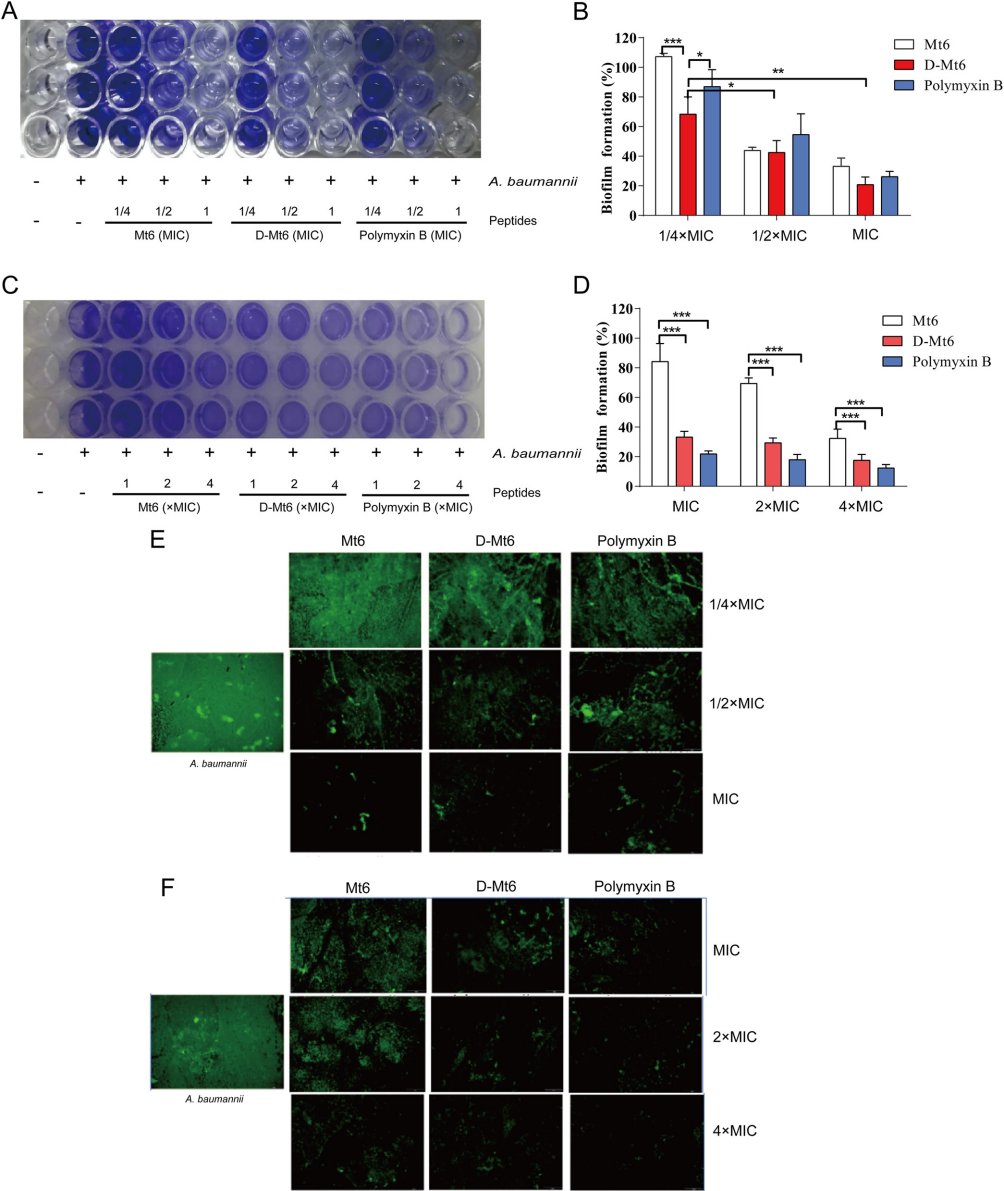
The derived peptides enhanced activity against bacteria in the biofilm mode of formation and eradication. (A) The Mt6-mediated or D-Mt6-mediated inhibition of biofilm formation was evaluated at the indicated concentrations via crystal violet staining. (B) Quantification of the proportion of biofilm formation analyzed in the assay, whose results are presented in panel A. (C) Effects of Mt6 and D-Mt6 at the indicated concentrations on the established biofilm eradication of A. baumannii using crystal violet staining. (D) The proportion analysis of panel C. (E) Representative fluorescence images by SYTO9 staining of A. baumannii residual biofilms pretreated with different concentrations of Mt6, D-Mt6, or polymyxin B, respectively. (F) Scavenging ability of different concentrations of AMPs on mature biofilm. *, *P* < 0.05; **, *P* < 0.01; and ***, *P* < 0.001; calculated using Student’s *t* test with GraphPad Prism.

Bacterial biofilm formation aids in disease pathogenicity and in the development of drug resistance. Therefore, the effects of D-Mt6 on biofilm formation and eradication were investigated. The results of a crystal violet staining experiment showed that the dark purple bacterial biofilm gradually fades in the presence of 0.25 × MIC, 0.5 × MIC, and the MIC of the derived peptides (Fig. 4A). Notably, at the concentration of 0.25 × MIC, the inhibitory effect of D-Mt6 was significantly better than that of Mt6 and was even stronger than that of polymyxin B (Fig. 4B). The preformed biofilm of A. baumannii was also reduced by D-Mt6 in a concentration-dependent manner (Fig. 4C and D). The residual quantities of A. baumannii biofilm after treatment with different concentrations of D-Mt6 were 33.26%, 29.31%, and 21.56%, respectively, which were similar to those of polymyxin B but significantly lower than the 84.26%, 69.43%, and 32.36% observed in the Mt6 treatment groups. A SYTO9 staining experiment observed via fluorescence microscopy also proved that the derived peptides could effectively inhibit the growth of A. baumannii biofilms while removing the established biofilms in a dose-dependent manner (Fig. 4E and F). The above findings indicate that the higher the concentration of D-Mt6 in the environment, the lesser the amount of bacterial biofilm formation. Meanwhile, the clearance effect of D-Mt6 on mature biofilm was significantly improved compared with that of Mt6.

### *In vivo* defensive effect of D-Mt6 and Mt6 against A. baumannii using a C. elegans model.

**FIG 5:**
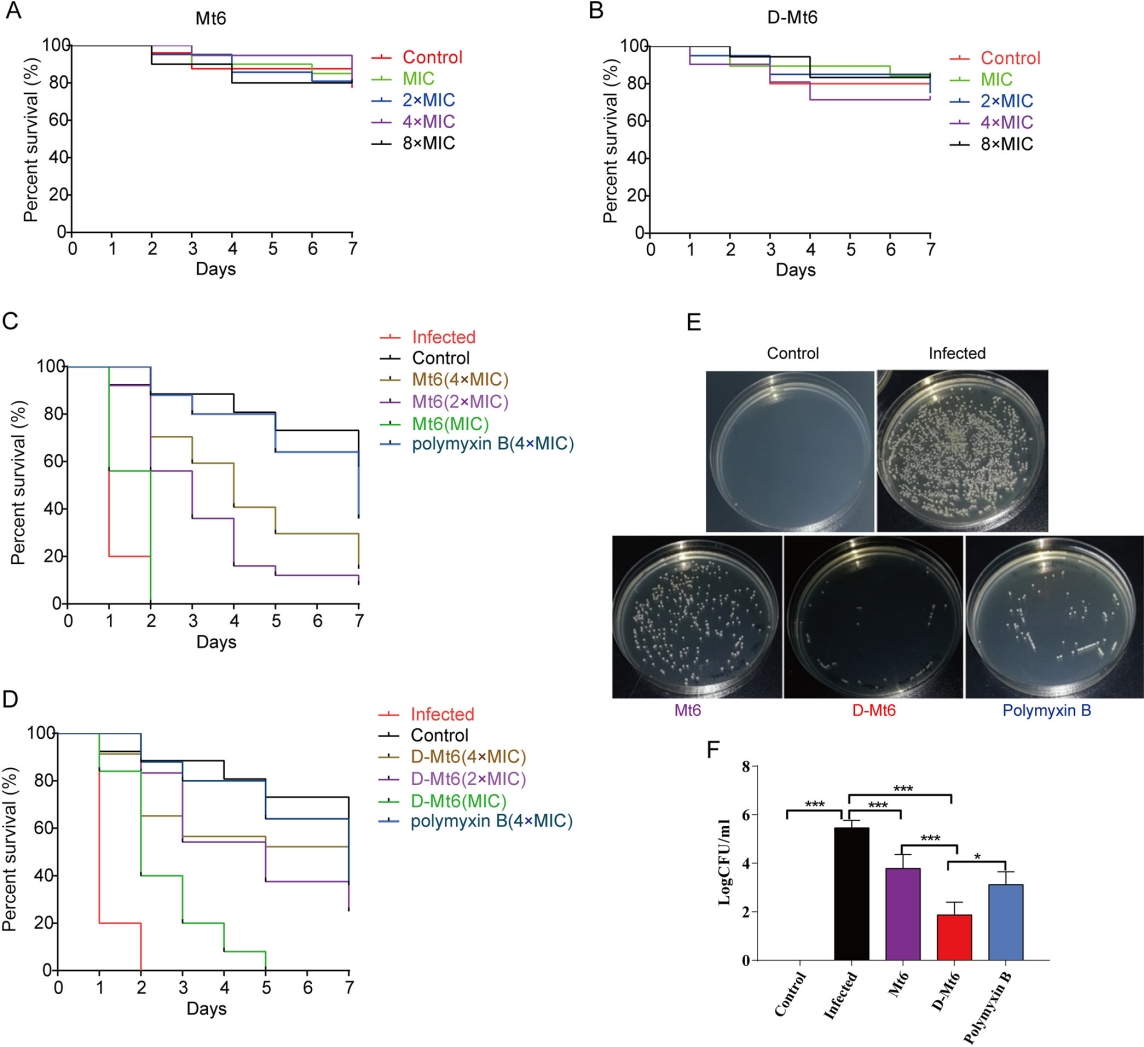
Effects of the derived peptides on the survival of A. baumannii-infected nematodes. (A) A toxicity study was initially conducted to determine the *in vivo* life span of the uninfected nematodes with Mt6 under different concentrations. (B) A toxicity study was initially conducted to determine the *in vivo* life span of the uninfected nematodes with D-Mt6 under different concentrations. (C) The effects of derived peptides at different concentrations on the viability of A. baumannii-infected C. elegans. (D) The calculated percentages of the effects of the derived peptides on the viability of A. baumannii-infected C. elegans with respect to the control. (E) The amounts of bacteria carried by A. baumannii-infected nematodes after 24 h of Mt6, D-Mt6, or polymyxin B treatment, respectively. (F) The statistics data of panel E. The control animals were not exposed to A. baumannii and were fed with E. coli OP50. The results represent the means of at least three independent experiments.

C. elegans has been employed as a model animal in antimicrobial sensitivity tests and in human pathogen studies. As an overture for experiments *in vivo*, a toxicity study about Mt6 and D-Mt6 was initially conducted against uninfected C. elegans. The nematode was judged dead when stiff without movement and response to touch (Fig. S3). As shown in Fig. 5A and B, no statistically significant life-span difference was oberved between the different concentrations of peptide-treated groups and the control group (without AMPs). This indicated that Mt6 and D-Mt6 had no toxicity to the nematodes at the test concentrations. Accordingly, the derived peptides were assessed for their effects on the viability of A. baumannii-infected C. elegans. Intriguingly, as displayed in Fig. 5C and D, all of the A. baumannii-infected nematodes died within 2 days. However, those pretreated with Mt6 or D-Mt6 followed by an A. baumannii infection displayed significantly increased worm survival in a concentration-dependent manner. Even the lowest dose of D-Mt6 could prolong the life spans of the nematodes. We observed the number of bacteria in the A. baumannii-infected nematodes with or without the derived peptides treatment. As shown in Fig. 5E and F, the number of bacteria in the nematodes was significantly reduced with Mt6 or D-Mt6 treatment compared with the infected group. Moreover, D-Mt6 treatment was more effective than Mt6 in reducing the number of bacteria, performing even better than the positive-control drug polymyxin B. These results indicated that both Mt6 and D-Mt6 had anti-A. baumannii activity *in vivo* and that the effect of D-Mt6 was superior to that of Mt6.

### Immunoregulatory activity analysis of AMPs targeting LPS.

**FIG 6:**
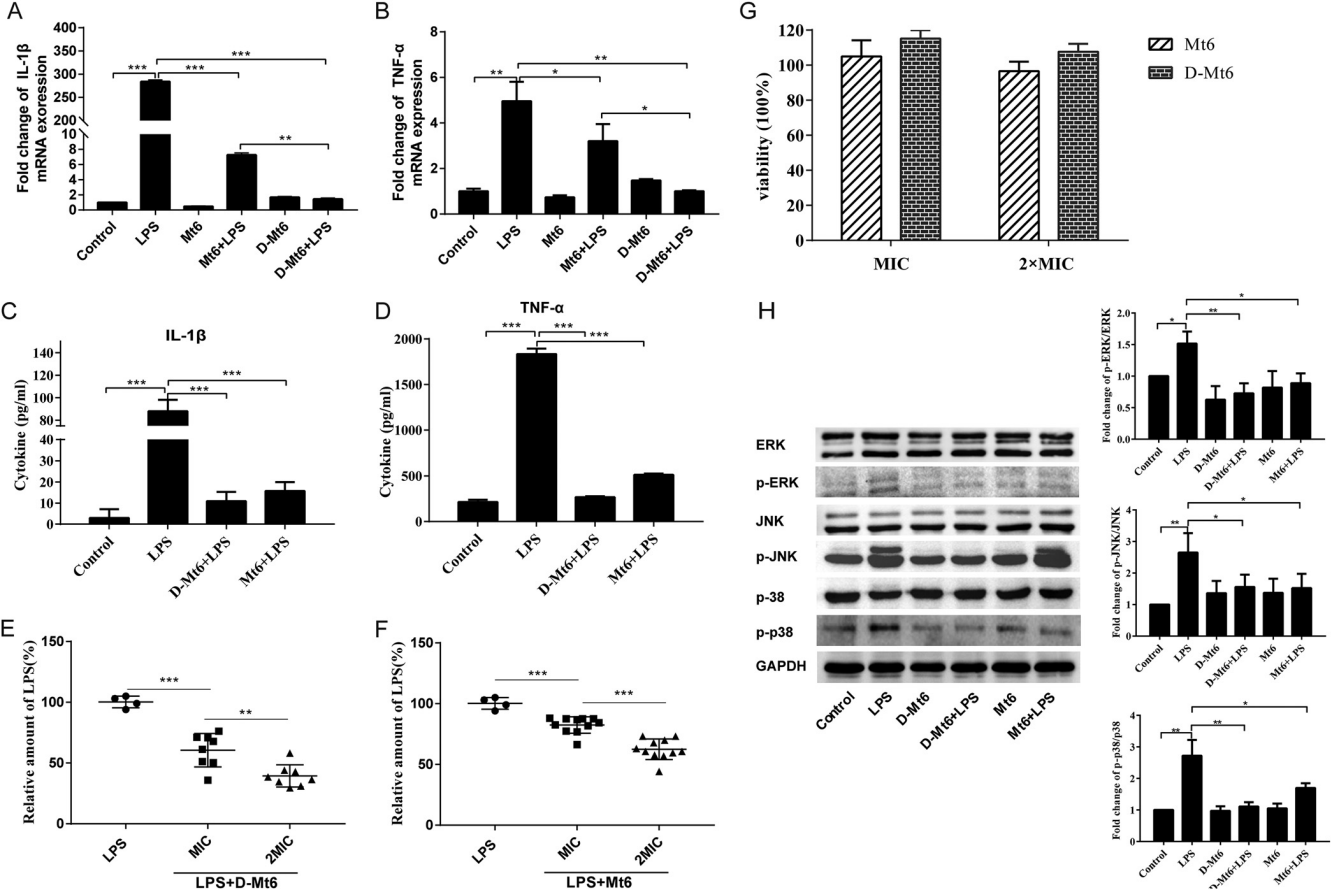
Mt6 and D-Mt6 inhibited LPS-induced inflammation in RAW 264.7 macrophages. (A) The effect of the MIC of Mt6 or D-Mt6 on IL-1β mRNA expression in RAW 264.7 macrophage cells with the presence of 100 ng/mL LPS for 24 h. (B) The effect of the MIC of Mt6 or D-Mt6 on TNF-α mRNA expression in RAW 264.7 macrophage cells with the presence of 100 ng/mL LPS for 24 h. (C) The levels of IL-1β cytokine in the supernatants of LPS-stimulated macrophages with or without Mt6 or D-Mt6 treatment were determined by enzyme-linked immunosorbent assay (ELISA). (D) The levels of TNF-α cytokine in the supernatants of LPS-stimulated macrophages with or without Mt6 or D-Mt6 treatment were determined by ELISA. (E and F) The neutralization abilities of different concentrations of D-Mt6 (E) and Mt6 (F) to LPS were examined by measuring the peptides’ inhibition of LPS-induced activation using an endotoxin detection kit. (G) CCK8 assay of Mt6 and D-Mt6 cytotoxicity against RAW 264.7 macrophages. The data represent the means ± standard errors of the means of *n* = 4 independent experiments. (H) The phosphorylation levels of ERK, JNK, and p38 protein were determined by Western blotting. The data were represented as mean ± standard error of the mean. (*n* = 3). Significant differences were detected via a one-way ANOVA and Newman-Keuls multiple comparison tests compared with cells that were incubated with 100 ng/mL LPS. *, *P* < 0.05; **, *P* < 0.01; and ***, *P* < 0.001.

LPS, as a bacterial endotoxin, is widely used in experiments to induce inflammation (32). In the present study, LPS-induced inflammation was employed in macrophages to evaluate the anti-inflammatory abilities of Mt6 and D-Mt6. The results revealed that the expression of TNF-α and IL-1β RNA were upregulated in LPS-induced RAW 264.7 macrophages, while they were conspicuously downregulated by both Mt6 and D-Mt6 pretreatment for 1 h (Fig. 6A and B). In line with the change of mRNA expression, the protein levels of TNF-α and IL-1β in the supernatant of LPS-stimulated macrophages were also decreased by Mt6 and D-Mt6 treatment (Fig. 6C and D). Thus, both Mt6 and D-Mt6 possessed suppressive effects against the proinflammatory response induced by LPS in macrophages, and the inhibition effect of D-Mt6 on the TNF-α release was more significant than the effect of Mt6 (Fig. 6D). Given the cationic and amphipathic nature of the derived peptides and that LPS is a negative charged molecule, the fact that the peptides reduced the LPS-induced inflammation is probably due to the neutralization effect. We further examined the neutralization ability of Mt6 and D-Mt6 to LPS using an endotoxin detection kit. The results showed that D-Mt6 could neutralize more than 40% of the endotoxin at the MIC and 68% at double the MIC (Fig. 6E) without affecting cell viability (Fig. 6F). However, Mt6 showed significantly reduced binding ability to LPS compared to D-Mt6, displaying only 17% inhibition of the endotoxin at the MIC (Fig. 6G). To further explore the protective mechanism of Mt6 and D-Mt6 in LPS-induced inflammation in macrophages, the expression of ERK, p38, and JNK was investigated by Western blotting. As displayed in Fig. 6H, the protein expressions of the three types of phosphorylated MAPKs (p-ERK, p-p38, and p-JNK) were significantly increased under LPS stimulation. Either Mt6 or D-Mt6 treatment significantly decreased the phosphorylation levels of ERK, p38, and JNK compared to LPS-stimulated cells.

## DISCUSSION

Drug-resistant infections have become more common in recent decades, necessitating the development of novel antibiotics. Unfortunately, as the prevalence of tigecycline resistance continues to rise, the therapeutic options against carbapenem-resistant infections remain limited (33), and colistin exhibits nephrotoxic effects after prolonged treatment (34–36). The housefly is a kind of vector insect with worldwide distribution. Due to the complex structure and diversity of pathogenic microorganisms in the breeding environment, its high resistance to diseases with AMPs as the effective molecule has attracted widespread attention. MAF-1 is a natural antimicrobial peptide that we first discovered in the hemlymphocytes of housefly larvae. After nearly 10 years of development, the new AMPs derived from MAF-1 present more activity and function (25, 37). However, the disadvantages, such as their low stability and the unknown antibacterial mechanism of the derived peptides, limit their clinical application as drug screening molecules. Based on the status quo, Mt6 and D-Mt6 as the two derived peptides were obtained by employing amino acid substitution and dextral transformation in the present study. D-Mt6 had high activity and good stability against A. baumannii. Moreover, it could inhibit the immune dysfunction induced by LPS. This study shows that D-Mt6 is a promising candidate drug derived from a peptide for use against A. baumannii.

Most bacteria have the capacity to form biofilms. The biofilms covering bacteria’s surfaces are more resistant to antibiotics (38, 39). AMPs seem to exhibit broad-spectrum activity and normally remain strongly effective against pathogens, even those in biofilm form (40, 41). Nonetheless, the antibacterial activity of AMPs frequently decreases or even inactivates based on their inherent defects, such as their reduced stability under physiological salt concentrations or low resistance to protease degradation (42–45). The salt resistance and anti-proteolytic properties of antimicrobial peptides have been improved using a variety of methods, including the substitution of noncanonical amino acids and d-amino acids (46–50). In the present study, the activity of both Mt6 and D-Mt6 against A. baumannii is not significantly affected in different ionic environments or PH environments, which was speculated to be caused by the increase of the charge number (from +2 to +9). Trypsin is an important hydrolytic enzyme in the digestive tract for the oral administration of antibiotics. As a result, the primary structure of antibacterial peptides is easy to hydrolyze and destroy, leading to a decrease or even a disappearance of antibacterial activity (51). Given this, the antimicrobial activity of derived peptides against A. baumannii in different concentrations of trypsin was measured. As anticipated, the D-Mt6 peptide is significantly more stable under the physiological environment of the enzyme compared to Mt6 (Table 2). Unfortunately, it remains unclear how d-enantiomerization affects the secondary structure and in turn causes the enhancement of stability. This requires a further study of small molecular peptides in terms of structural assessment.

The mechanism of AMPs resistance to Gram-negative bacteria is usually due to their membrane-breaking action (52). Electron microscopy and SY9/PI double-staining confocal microscopy were employed to observe the effect of the derived peptide on bacterial membranes. The results showed that the cell contents of A. baumannii were released in large quantities after 1 h of treatment with D-Mt6, which resulted in the complete disintegration, death, and damage of the bacteria. Furthermore, D-Mt6 exert bactericidal activity in 10 min after coculturing with the bacteria and effectively maintain it for at least 12 h, significantly faster than the clinical drug polymyxin B performs. In addition, when combined with traditional antibiotics, it presented a synergistic interaction between D-Mt6 and tobramycin as well as an additive effect in combination with polymyxin B. Studies found that the addition of d-amino acids could augment the transmembrane delivery of peptide (53, 54). Hence, the presence of D-Mt6 may enhance the permeability of the bacterial cell membrane, thereby facilitating the traditional antibiotics more easily entering the bacteria to perform their anti-bactericidal functions. Because systemic drug administration is known to result in lower sputum concentrations than aerosolized treatment, the potentiating effect of D-Mt6 may be advantageous when delivering tobramycin intravenously.

In clinical settings, A. baumannii is prone to form biofilms on solid surfaces and cause infection (55). The drug resistance of biofilm is often more than 1,000 times higher than that of free bacteria (56), which exempts bacteria from host defense systems or antimicrobial clearance. Here, we found that both D-Mt6 and Mt6 had the activity of inhibiting the formation of an A. baumannii biofilm with a dose-dependent response, and D-Mt6 possessed better resistance than did Mt6. Moreover, we also observed the efficacy of D-Mt6 against mature biofilms (48 h growth) of A. baumannii. Undoubtedly, this ability to resist biofilms makes D-Mt6 more likely to become a potent anti-A. baumannii infection agent.

A key aspect of developing new antibacterial drugs is their effectiveness *in vivo*. To evaluate the abilities of D-Mt6 *in vivo*, the bacterium-infected C. elegans was employed. As a model organism, C. elegans could avoid the shortcomings of a mammalian model (expensive and time-consuming) and reduce the cost of drug screening, leading to it being widely used in large-scale drug screening (57, 58). In the present study, the toxicity of D-Mt6 and Mt6 in nematodes was first evaluated. Neither D-Mt6 nor Mt6 affected the growth of nematodes at the maximum test dose (8 × MIC). Moreover, D-Mt6 has more significant and powerful effects on prolonging the survival time of A. baumannii-infected C. elegans and on reducing the number of bacteria carried by nematodes than does Mt6. This indicates that D-Mt6 has a more stable internal environment in animals than Mt6.

When certain antimicrobial drugs kill Gram-negative bacteria, LPS is liberated into the bloodstream. Oligomers aggregated by LPS will stimulate the macrophages and monocytes to release large proinflammatory cytokines. In turn, cytokines play a role in the pathophysiology of septic shock and other immunological disorders (23, 59–61). As a result, much emphasis has been placed on the development of novel cationic AMPs with antimicrobial and LPS-neutralizing properties. We investigated the effects of Mt6 and D-Mt6 peptides on LPS-induced macrophage inflammation in this recent study. The findings revealed that both Mt6 and D-Mt6 significantly inhibited TNF-a and IL-1β secretion as well as their mRNA expression induced by LPS in RAW 264.7 macrophage cells. These observations and the positive charge properties of cationic AMPs might correlate with the striking ability of D-Mt6 to neutralize the activity of LPS established in the Limulus amebocyte lysate assay (Fig. 6), which is one of the most widely used methods for testing LPS-binding activity (62). MAPK signaling is one of the classic inflammatory cytokine release pathways (63), and the inhibition of MAPK signaling further confirmed that the anti-inflammatory property of Mt6 and D-Mt6 peptides is linked to LPS neutralization. LPS significantly upregulated the phosphorylation of ERK, p38, and JNK, while cells treated with Mt6 and D-Mt6 exhibited dampened levels (Fig. 6). These findings propose that the intracellular anti-inflammatory effect of Mt6 and D-Mt6 on the MAPK signaling pathway plays a key role in LPS-induced inflammation.

While we have demonstrated that the binding of D-Mt6 to LPS on the surface of A. baumannii results in the disruption of the outer membrane, it remains unclear how this leads to cell lysis and death. It is hypothesized that damage to the LPS monolayer causes polymyxin to traverse the outer membrane through a process of “self-directed uptake”, though this has not been confirmed experimentally. The mechanism, employed by colistin, of damaging the cell membrane is also unclear (64–66). The sterilization ability of the derived peptides in this study is also possible through the same mechanism as polymyxin, which is still being researched. Although we have provided evidence that our peptides possess LPS neutralizing activity and exert anti-LPS induced inflammation by regulating intracellular immune signaling, the specific target of the peptide against LPS remains unclear at the present time. It has been deduced that the possible anti-LPS mechanisms of peptides mainly consist of two aspects. First, AMPs directly antagonize LPS by inhibiting the anionic amphiphilic composition of LPS such as lipid A domains (67). Second, AMPs competitively block the binding of the LPS-LPB complex to the CD14 receptor on immune cells, thus making it inaccessible for the TLR-4 receptor, and then block the activation of intracellular proinflammatory signals (68, 69). Our findings suggest that Mt6 and D-Mt6 neutralize LPS-induced proinflammatory responses, which results not only from it directly extinguishing LPS due to its strong cationic properties and hydrophobicity but also from its inhibitory effect on the MAPK signaling pathway. Therefore, it is imperative to carry out studies in greater depth to demonstrate the multitarget functions of our peptides in anti-inflammatory activity.

In summary, we successfully designed Mt6 and D-Mt6 with a broad-spectrum antimicrobial activity, especially against *A. baumannii*, based on MAF-1A. Both of them have synergistic or additive effects in amalgamation with conventional antibiotics, stability, an anti-biofilm property, and an anti-LPS induced inflammatory response. Our results indicate that Mt6 and D-Mt6 kill *A. baumannii* bacteria by damaging the membrane integrity. Meanwhile, they are also able to discourage the formation of new biofilms and eliminate preformed *A. baumannii* biofilms. However, D-Mt6 protects against LPS induced inflammation through its neutralization of LPS and suppression of MAPK signaling in macrophages. Overall, this study suggests that D-Mt6 could be an exciting molecule to use as a starting point for developing a novel anti-*A. baumannii* and anti-inflammatory agent.

## MATERIALS AND METHODS

### Materials and bacterial strains.

Two strains of Gram-positive bacteria (Staphylococcus aureus [ATCC 25923] and Bacillus subtilis [ATCC 6633]), four strains of Gram-negative bacteria (Escherichia coli [ATCC 25922], Klebsiella pseudomonas [ATCC 700603], Pseudomonas aeruginosa [ATCC 27853], and Acinetobacter baumannii [ATCC 19606] and fungus Candida albicans [ATCC 10231]) were obtained as gifts from the Pathogenic Biology Laboratory of Guizhou Medical University. Primary antibodies of phospho-ERK, ERK, phospho-JNK, JNK, phospho-p38, p38, and GAPDH were purchased from Cell Signaling Technology in the USA. The LIVE/DEAD BacLight Bacterial Viability Kits and the TNF-a/IL-1β ELISA kits were procured from Thermo Fisher Scientific in the USA. The polymyxin B, tobramycin, Mueller-Hinton Broth Medium (MHB), Luria-Bertani Medium (LB), Trypsin, Sabouraud's Glucose Broth Medium, and Brain Heart Infusion Medium (BHI) were purchased from Suolaibao, China. The Limulus amebocyte lysate assay kit was purchased from Xiamen Tachypleus Amebocyte Lysate Reagent, China. The wild-type Caenorhabditis elegans (Bristol strain N2) was obtained as a gift from the Department of Genetics of Xuzhou Medical University. The other reagents used in this study were supplied from Beyotime, China.

### Peptide synthesis.

All peptides synthesized for this study were synthesized from GL Biochem Ltd. (Shanghai, China) and purified via high-performance liquid chromatography (HPLC) to confirm their purity as greater than 95%.

### Antimicrobial assay.

The peptides’ antibacterial activity against the aforementioned pathogens was assessed by determining the MIC. In brief, the bacteria were inoculated and cultured to the mid-log-phase in fresh Luria-Bertani broth (LB) at 37°C. In Mueller-Hinton broth (MHB), the bacterial inoculum suspensions were adjusted and diluted to 1.5 × 10^6^ CFU/mL, while C. albicans was cultivated in Sabouraud's Glucose Broth Medium (SDB) at 37°C. The peptides or polymyxin B were 2-fold serially diluted to obtain concentrations ranging from 0 to 256 mg/mL. Subsequently, different doses of peptides were added to each well of a sterile 96-well plate containing inoculum suspensions, and the plate was incubated at 37°C for 24 h. The MIC was defined as the lowest concentration of chemicals that prevented apparent turbidity upon visual inspection. Furthermore, the peptides’ MICs were measured in the presence of various physiological conditions. The 1.5 × 10^6^ CFU/mL of A. baumannii were treated with peptides in MHB supplemented with various concentrations of salts (150 mM NaCl, 4.5 mM KCl, and 1 mM MgCl_2_), 0.15 mg/mL Trypsin, 0.6 mg/mL Trypsin, or different pH environments (pH 5.45, pH 7.45, pH 9.45). All experiments were done in triplicate.

### Time-killing kinetics assay.

The broth microdilution method was used to perform time-killing assays per the Clinical and Laboratory Standards Institute guidelines. In brief, mid-log-phase *A.baumannii* were adjusted to 1.5 × 10^6^ CFU/mL in MHB media and treated for 0 to 12 h at 37°C with peptides and antibiotics at the MIC. At each exposure time, aliquots of the mixture were diluted with fresh MHB media before plating 100 μL of the diluted bacterial suspension onto MH agar plates to determine viability counts. The colonies were enumerated, and killing curves were created by plotting the log_10_ CFU/mL after 1 or 12 h of incubation at 37°C.

### Electron microscopy.

Mid-log-phase bacteria A. baumannii grown in LB broth were adjusted to a density of 1.5 × 10^6^ CFU/mL, and peptides were added to the bacterial suspension at the MIC and incubated at 37°C under constant shaking for 1 h. 3% glutaraldehyde in 0.1 M phosphate buffer (pH 7.4) was used to fix portions of bacteria cultured with or without peptides for 2 h at 25°C, which were centrifuged at 2,000 rpm. After 30 min of washing, samples were postfixed for 1 h with 2% osmium tetroxide and washed three times with distilled water for 15 min. After standard ethanol dehydration, samples were freeze-dried, mounted to a stub, and coated with platinum using an ion sputter (60, 70, 80, 90, 95, and 100%). A scanning electron microscope (FEI, USA) was then used to examine the images.

### Confocal laser scanning microscopy of A. baumannii.

An A. baumannii suspension with 0.5 maltensite concentration was prepared, followed by the addition of Mt6 and D-Mt6 with a final concentration of the MIC and the addition of polymyxin B as a positive-control. They were then cultured at 37°C for 1 h. After that, the medium was centrifuged at 12,000 rpm/min. After the resuspension of PBS, propidium iodide (PI) with a final concentration of 5 μg/mL and SYTO9 with a final concentration of 5 μmol/mL were added and subsequently incubated for 30 min away from light. It was then washed 3 times with PBS. Next, it was resuspended in 50 μL of PBS, and then 20 μL of the resuspension bacterial solution was blotted on slides (after poly-lysine treatment). According to the LIVE/DEAD BacLight Bacterial Viability Kit, the bacteria stained red by PI were considered dead, while the bacteria stained green by SYTO9 were considered alive under the confocal microscope.

### Checkerboard assay.

The combination effect of peptides with polymyxin B or tobramycin on A. baumannii was evaluated using a checkerboard assay on a 96-well polypropylene plate. Briefly, each well contained 100 μL of Mueller-Hinton medium, differing concentrations of antibacterial agents, and an inoculum of 1.5 × 10^5^ CFU per well of A. baumannii. The 96-well checkerboard assay was incubated for 24 h at 37°C. The fractional inhibitory concentration index (FICI) was calculated as follows:

FICI = [(MIC of peptide in combination)/(MIC of peptide alone)] + [(MIC of antibiotic in combination)/(MIC of antibiotic alone)]

### Anti-biofilm activity.

The anti-biofilm activity of Mt6 or D-Mt6 peptide on A. baumannii was assessed as formally described, with many modifications (70–72). Briefly, to assess the ability of peptides on the inhibition of biofilm formation, pregrown A. baumannii was added to peptide solutions at different concentrations ranging from 1 to 4 μg/mL in MHB at a final concentration of 1.5 × 10^6^ CFU/mL. Following incubation for 24 h at 37°C, the planktonic bacteria were removed by washing with sterile PBS solution three times. The biofilm was then fixed with methanol for 15 min, air-dried at room temperature, and stained for 5 min with 0.1% crystal violet. After rinsing the wells with water, 100 mL of 95% ethanol was added to each well, and the plate was shaken for 30 min at room temperature. A measurement of absorbance was performed at 595 nm using a microplate reader (Boten Instrument Co., USA) to determine the biofilm biomass. The preformed biofilm reduction activity was also evaluated. The biofilm of *A. baumanii* was cultured in a 96-well plate by adding 100 μL of bacteria (1 × 10^6^ CFU/mL) for 24 h at 37°C. The wells containing biofilm were rinsed three times with PBS. Serial dilutions of the Mt6 or D-Mt6 peptide (1 to 4 × MIC) were prepared in a fresh 96-well plate, and 100 μL of the suspension were transferred to the biofilm-containing plate. The plates were then incubated for 24 h at 37°C. Following incubation, the wells were emptied and washed three times with PBS before being air-fixed for 1 h in an aseptic environment. As indicated above, the percentage of biofilm clearance was determined using a crystal violet assay. Each assay was carried out three times. We also used the SYTO9 dye to observe the composition and morphology of the biofilm. Following the addition of *A. baumanii* cells to each well, 100 μL of SYTO9 working solution was added, and the plates were incubated on a rocking table in the dark. The fluorescence was then measured using a fluorescence microscope (Olympus, Japan).

### *In vivo* efficacy of peptides in a C. elegans infection model.

A toxicity assay was first carried out to assess nematode survival rates in the presence of various concentrations of Mt6 or D-Mt6. The L1 worms were grown to the L4 stage in E. coli lawns, and the killing assay was carried out at room temperature by inoculating 20 L4 nematodes per NGM plate that already contained the peptides (or E. coli OP50 as a control). The survival of the nematodes was observed by microscopy every 24 h and counted continuously for 7 days. Killing assays were repeated three times, and the results of a representative experiment were displayed. The A. baumannii infection and the peptide treatments of C. elegans were performed as initially described (73, 74), with the following alterations. In short, the worms were fed E. coli OP50 for 24 h in NGM plates before being transferred to A. baumannii-seeded NGM plates. After infection, the worms were collected and transferred in triplicate to microcentrifuge tubes (for survival assessment) or 96-well plates (for bacterial CFU determination) with Mt6 or D-Mt6 (at 1 to 4 × MIC) treatment. The number of dead worms were counted each day until the 7th day of incubation. To assess the bacterial load in the worms, A. baumannii-infected worms with or without a peptide treatment were first rinsed three times with PBS and then lysed via grinding. After diluting the solution appropriately, the plate was painted and incubated at 37°C for 24 h before counting. Uninfected worms served as a negative-control.

### Cell culture and cytotoxicity assay.

RAW 264.7 murine macrophage cells were cultured in Dulbecco’s modified Eagle’s medium with 10% fetal bovine serum and penicillin-streptomycin. We assessed the cytotoxic effects of peptides using the CCK-8 assay (75). In brief, in 100 μL medium, polystyrene 96-well plates were seeded with 1 × 10^5^ cells per well. After 12 h of incubation at 37°C in 5% CO_2_, cells were exposed to peptides at 2-fold concentration increments for 24 h, and then 10 μL of CCK-8 was added to each well and incubated for 2 h. Absorbance measurements were performed using a microplate reader at 450 nm. Three replicates were performed for all of the peptide concentrations.

### Quantitative reverse-transcription real-time polymerase chain reaction (qRT-PCR).

RAW 264.7 macrophage cells were cultured in a 6-well plate to 70 to 80% confluence and then treated with peptides at the MIC for 1 h. After 1 h of incubation, the cells were stimulated with LPS from E. coli O111:B4 (100 ng/mL) in the presence or absence of peptides for 12 h. The samples were divided into six groups: vehicle (control), LPS (100 ng/mL), LPS + D-Mt6, D-Mt6 alone, LPS + Mt6, and Mt6 alone. According to the manufacturer's instructions, total RNA was isolated from cells using the TRIzol reagent. A spectrophotometric analysis confirmed the purity and integrity of the RNA. Using the High-Capacity cDNA Reverse Transcription Kit, 1 μg of total RNA was reverse transcribed to provide complementary DNA (cDNA). qRT-PCR was used to amplify the cDNA using SYBR Select Master Mix, according to the manufacturer's instructions. Triplicate samples were analyzed (*n* = 3 independent experiments). PCR amplification was carried out for initial denaturation at 95°C for 5 min, followed by 40 cycles of denaturation at 95°C for 15 s, annealing for 15 s at 55°C, and extension for 15 s at 72°C.

The primers used for the amplification were:

forward 5′-TGTGTTTTCCTCCTTGCCTCTGAT-3′ and reverse 5′-TGCTGCCTAATGTCCCCTTGAAT-3′ (*IL-1β*);

forward 5′-CCACCACGCTCTTCTGTCTACTG-3′ and reverse

5′-GCCATAGAACTGATGAGAGG-3′ (*TNF-α*);

forward 5′-CGTGGGCCGCCCTAGGCACCA-3′ and reverse

5′-TTGGCCTTAGGGTTCAGGGGGG-3′ (*β-actin*).

### Quantification of inflammatory cytokine production in LPS-stimulated macrophage cells.

RAW 264.7 cells were seeded in 12-well plates for 24 h and then treated with peptides for 1 h at 37°C. The cells were then stimulated with LPS for 24 h in the absence or presence of peptides. After incubation, an enzyme linked immunosorbent assay (ELISA) was used to detect the levels of inflammatory cytokines (TNF-α and IL-1β) using the culture supernatant. The absorbances were measured with a microplate reader at 450 nm per the manufacturer’s instructions. The assay was conducted in triplicate.

### Western blot.

The protein content of all experimental groups of RAW264.7 cells was extracted using RIPA lysis buffer that was suitably combined with a mixture of protease inhibitors and phosphatase inhibitors. Then, the cells were scraped and placed in microcentrifuge tubes. The homogenate was placed on ice and sonicated 4 times for 5 s at intervals of 6 s. This was followed by centrifugation at 12,000 × *g* for 20 min at 4°C. A BCA Assay Protein Assay Kit was used to measure the protein concentration in the supernatant (Thermo Fisher Scientific, Inc., MA, USA). The same amounts of protein were separated by sodium dodecyl sulfate-polyacrylamide gel electrophoresis (SDS-PAGE) and then electro-transferred to a PVDF membrane. After blocking the membranes with 5% nonfat milk for 1 h, they were incubated overnight with the appropriate primary antibodies (1:1000 dilution) at 4°C. This was followed by incubation with HRP-inked anti-rabbit IgG secondary antibody or HRP-linked anti-mouse IgG secondary antibody for 2 h at room temperature. The detection of protein bands was performed using Clarity ECL Western blot substrate (Bio-Rad) and then visualized with a ChemiDoc Touch imaging system (Bio-Rad).

### LPS neutralization assay (TAL Assay).

The ability of Mt6 or D-Mt6 to neutralize LPS was assessed by a commercially available chromogenic Tachypleus Amoebocyte Lysate (TAL) Kit (Xiamen Horseshoe Crab, Inc., China). In a 96-well plate, the MIC or 2 × MIC concentrations of peptides were incubated for 1 h at 37°C with 1 unit of endotoxin, and then 100 μL of TAL reagent was added and incubated for another 15 min. This was followed by the addition of 100 μL of a chromogenic substrate. After a 10-minute incubation period, the reaction was stopped by the addition of 25% acetic acid, and the amount of colored product released was quantified spectrophotometrically at 545 nm. The percentage of LPS binding was assessed as follows: ([OD LPS–OD peptide] / OD LPS) × 100%. All assays were repeated twice.

### Statistical analysis.

The experimental data were presented as mean ± standard deviation (SD). The data were analyzed using a Student’s *t* test or a one-way analysis of variance for comparisons. The survival curve was tested via log-rank. The SPSS 19.0 statistical software package was used for data processing. A *P* value of <0.05 was considered indicative of a statistically significant result.
