# Supplementary material for: Antimicrobial and Anti-Inflammatory Activities of MAF-1-Derived Antimicrobial Peptide Mt6 and Its D-Enantiomer D-Mt6 against Acinetobacter baumannii by Targeting Cell Membranes and Lipopolysaccharide Interaction
Source: Microbiol Spectr. 2022 Oct 3;10(5):e01312-22. doi: 10.1128/spectrum.01312-22 (PMC9603722; doi:10.1128/spectrum.01312-22)
Supplement: Supplemental file 1 — Fig. S1 and S2. Download spectrum.01312-22-s0001.pdf, PDF file, 0.5 MB [file spectrum.01312-22-s0001.pdf]

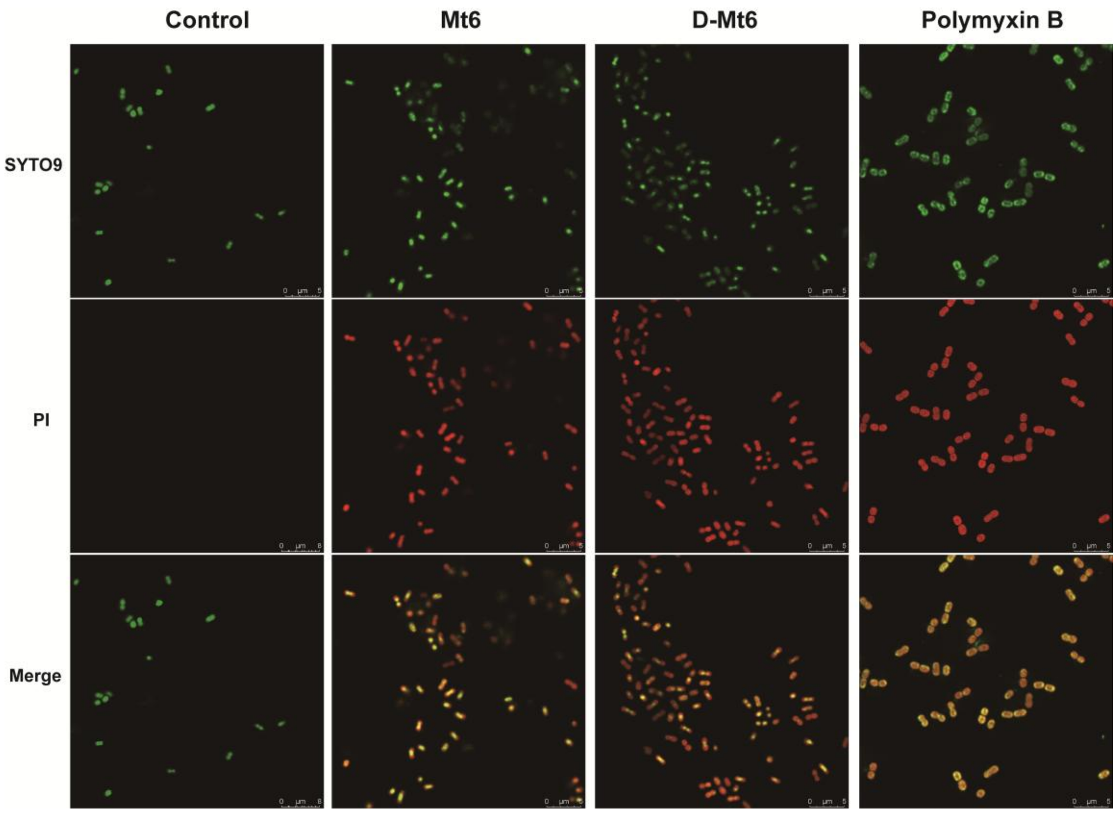

Fig. S1 Representative confocal fluorescence images of *A. baumannii* cells with SYTO 9/PI stains by the LIVE/DEAD BacLight Bacterial Viability Kits. Live and dead cells stained by SYTO9 and PI are presented as green and red fluorescence, respectively. Scale bars: 5 μm.

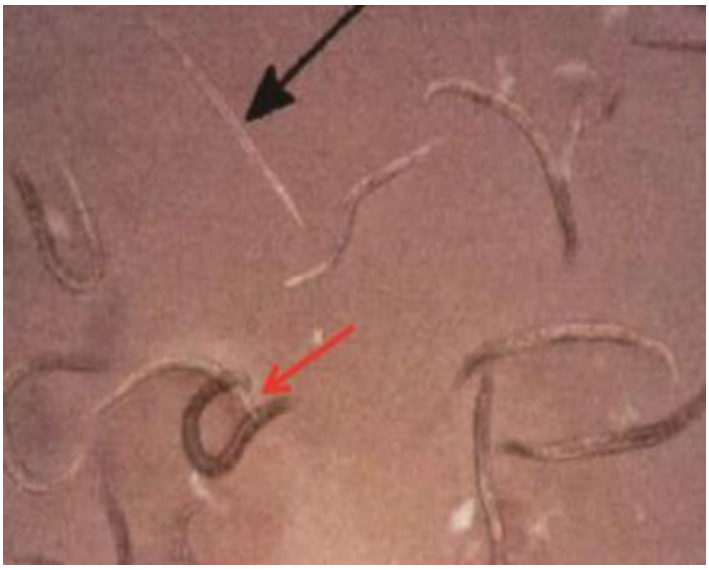

Fig. S2 The survival/death status of *C. elegan*s under the microscope. The red arrow is the survival form of *C. elegans*, the black arrow is the death state of *C. elegans* (×40).
